# In-Game Workload Demands of Position Players in Major League Baseball

**DOI:** 10.1177/19417381231179970

**Published:** 2023-06-16

**Authors:** Jonathan Freeston, Lonnie Soloff, Mark Schickendantz, Jason Genin, Salvatore Frangiamore, Rod Whiteley

**Affiliations:** † Exercise, Health and Performance Research Group, The University of Sydney, Australia; ‡ Cleveland Guardians Baseball, Cleveland, Ohio; § Cleveland Clinic, Ohio; ‖ Aspetar Orthopaedic and Sports Medicine Hospital, Doha, Qatar

**Keywords:** athletic training, baseball, injury prevention, physical therapy/rehabilitation

## Abstract

**Background:**

Athletes who are well prepared for the physical demands of competition are less susceptible to injury. Defining and then preparing athletes for these in-game demands is critical to athlete health and performance. The injury burden within Major League Baseball (MLB) is significant and differs by position. Despite its importance, the workload demands have not been described for position players in MLB.

**Hypothesis:**

That running demands would be significantly higher for outfielders, followed by infielders, and catchers, respectively, while batting and baserunning metrics would be similar across positions.

**Study Design:**

Cohort study.

**Level of Evidence:**

Level 3.

**Methods:**

Total and high-speed running distance (>75% Vmax), high-speed running count, hard accelerations (>2.78 m/s/s), defensive and baserunning minutes, total and hard throws (>75% max), and bat swing counts were calculated from Statcast data. Players with 100 games or more in the 2018 season (*n* = 126) were included for analysis.

**Results:**

All offensive and baserunning metrics were similar across positions; however, significant positional differences were observed for defensive and overall workload metrics. High-speed running was highest among outfielders (*F*_1,7_ = 27.1, *P* < 0.01), followed by infielders, then catchers. Hard accelerations (*F*_1,7_ = 12.9, *P* < 0.01) were highest among first basemen, then outfielders, remaining infielders, and catchers. Total throws (*F*_1,7_ = 17.7, *P* < 0.01) were highest among middle infielders. Hard throws (*P* < 0.01) were highest among shortstops and third basemen.

**Conclusion:**

In-game workloads differ significantly by defensive position in MLB. These differences in running, throwing, and hitting volumes have significant implications for physical preparation and injury return-to-play progressions to optimize performance and minimize injury and reinjury risk for these athletes.

**Clinical Relevance:**

These data provide insight into how best to prepare athletes of different positions for the demands of the game both in terms of preseason preparation as well as return-to-play benchmarks following injury. These data should also serve as a platform for future research into the relationship between workload and injury among professional baseball players.

Major League Baseball (MLB) is commonly regarded as having among the most demanding game schedules in all professional sports. In addition to approximately 7 weeks of preseason work, the regular season features 162 games across a 6-month period at an average of 6.3 games per week. This presents a unique challenge for physical preparation, athlete monitoring and return to play after injury.

The injury burden throughout MLB is significant and has been well documented previously.^10^ Between 1998 and 2015, the number of players (pitchers and position players combined) on the injured list (IL) each year ranged from 387 to 536, resulting in a total duration of between 21,132 and 30,302 IL days annually, costing between $136,397,147 US and $694,835,359 US annually for the injured player and their replacement combined.^10^ While these numbers may be slightly inflated due to “phantom” injury designations for roster management purposes, they represent a significant economic impact on the players and their organizations.

In a study of injuries between 2011 through 2016, pitchers bore the highest injury burden (39.1%), followed by infielders (27.1%), outfielders (22.8%), catchers (11.0%), and designated hitters (0.1%).^4^ Whereas injuries to the upper body are common (shoulder 15%, elbow 9%), significant burden also results from injuries to the lower limbs (upper thigh 12%, knee 6%, ankle 5%), many of which are soft tissue injuries.^4^ Notably, hamstring strains are the single most common reason for time loss among MLB and Minor League Baseball players.^1,4,19^

Given the significant injury burden experienced by MLB players, a thorough understanding of the workload demands of professional baseball is needed to facilitate appropriate workload management for healthy and injured athletes. Workload management is a key component of sports injury prevention,^11^ facilitating opportunities for injury prevention during preseason preparation, in-season management, and rehabilitation return to play, respectively.

Recent evidence suggests that athletes who are best prepared for the demands of their sport are at a reduced risk of injury. This preparation involves both the appropriate exposure to workload demands as well as the development of key physical capacities to build resiliency. Specifically, it has been shown repeatedly across multiple sports that athletes who have been exposed to training volumes similar to those encountered in gameplay are less likely to sustain injury.^6,13,14,18^ Athletes who have been exposed to high chronic loads and/or completed more preseason training sessions have been shown to experience lower injury risk.^12,14,17,22^ Conversely, athletes with minimal exposure to key workload demands or those that experience sudden increases in workload (such as those that occur when an underprepared athlete transitions from the preseason to the regular season) have shown increased injury risk.^8,9,15^ In the rehabilitation context, high running workloads after lower limb muscle injury delay return-to-play timelines but protect against subsequent injury.^20^ Importantly, the development of physical capacities such as strength, speed, repeated-sprint ability and fitness can mitigate the risk of injury.^16,17^

Therefore, an understanding of typical in-game demands for baseball players is important for sports medicine practitioners to (1) ensure appropriate physical preparation is achieved in spring training to minimize the risk of injury in the early parts of the season, (2) modify in-season workloads in response to in-game demands, and (3) determine objective return-to-play criteria after injury and ensure athletes are adequately prepared for game demands.^3^

Previous research involving workload demands of position players within professional baseball has been limited. Only one study to date has explored the relationship between lower limb soft tissue injuries (hamstring and calf strains) and workload among position players at the professional level,^7^ during which they showed a link between injury and workload factors such as fewer days rest, increased innings, and increased plate appearances. Workload (defined as games played, at-bats, plate appearances, and innings) was not associated with changes in hip range of motion throughout the course of a season of professional baseball.^5^ However, the specific workload demands were not quantified in either of these studies in terms of external load factors such as running distance or intensity, or hard accelerations. One study to date has quantified the number of throws made by players of different positions; however, this involved players at the collegiate level.^2^

No study to date has quantified the workload of position players within MLB in terms of the amount of throwing, hitting, or running activity. Given the diverse injury profile of professional baseball players, specifically the burden of lower limb soft tissue injuries, a thorough understanding of the workload demands of MLB players is needed.

This study seeks to describe the position-specific physical demands of MLB players to improve physical preparation (including conditioning strategies) and better monitor return to play after injury. In addition to defining the demands of a single game, given the uniquely dense playing schedule, we also sought to define the demands of a typical week as well as the worst case 7-day period an athlete could reasonably be exposed to. This was done to capture the cumulative effects of multiple games within a calendar week.

We hypothesized that running demands would be significantly higher for outfielders, followed by infielders, and catchers, respectively, while batting and baserunning metrics would be similar across positions.

## Methods

### Sample

This cohort study was performed with the approval of the MLB Research Committee. Study data were analyzed from Statcast data provided by MLB to each of the participating clubs. The Statcast system is installed in each MLB stadium and consists of 2 camera banks typically located on the third base line. The system tracks individual players at 30 Hz with an onfield resolution of 0.005 to 0.076 m per pixel. All professional baseball players who participated in the 2018 season were eligible for inclusion in the study; however, only those that participated in >100 games at the same position were included in the final analysis. Only games played at the player’s primary position were included in the analysis. A total of 126 players (48.9% American League, 51.1% National League) across 30 different teams met the inclusion criteria with the following positional distribution: 18 first-basemen, 18 second-basemen, 20 third-basemen, 21 shortstops, 12 catchers, 16 center-fielders, 10 left-fielders, and 12 right-fielders. This yielded 17,175 total games at an average of 127.2 ± 16.3 games per player, which is equivalent to 78.5% of all available games.

### Dependent Variables

The following variables were generated from the raw Statcast data and analyzed:

Total running distance was defined as the distance in meters covered by each player regardless of intensity, whereas high-speed running distance was defined as the distance in meters covered by each player above 75% of their maximum recorded running speed. We defined maximum running speed as the 95th percentile of the player’s maximum running values across the entire season. High-speed running count was defined as the number of times a player went above 75% of their maximum running speed. Hard acceleration count was defined as the number of times a player accelerated at greater than 2.78 m/s^2^. Minutes baserunning and defensive minutes were defined as the total time spent on the basepaths and the number of minutes spent in the field, respectively. Total throws were defined as a count of all throws made in a game regardless of intensity, whereas hard throws were defined as the count of throws made above 75% of the player’s maximal throw speed recorded during the 2018 regular season. Bat swings were defined as the sum of all in-game swings made by each player irrespective of contact with the ball. Notably, given that Statcast data capture events only when the ball is in-play, all metrics represent in-play values only, and do not reflect any player movements or activities outside of play. For example, total running distance does not reflect distance covered running to and from a player’s position at the start and end of an inning; throws do not reflect warm-up throws or those made after the completion of an out; and swings do not reflect warm-up swings.

### Statistics

A game was defined as the mean and standard deviation for each metric across all games for all players from a given position. A typical 7-day period was calculated by first breaking the regular season into consecutive 7-day periods for each player. The workload demands within each 7-day period were then summed. The mean and standard deviation for each 7-day period was then calculated for each player from a given position. Data were assessed for normality using Shapiro-Wilk test. Between-group (position) differences for normally distributed data were assessed using analysis of variance with the Bonferroni adjustment applied. Between-position differences for non-normally distributed data was assessed using the Kruskal-Wallis test. The criterion alpha was set at α < 0.05.

### Results

Workload data for a typical game and a typical 7-day period of MLB are presented below (Tables 1 and 2). While all offensive and baserunning metrics were similar across positions, significant positional differences were observed for defensive and overall workload metrics despite similar time on defense and similar time on the basepaths across the positions.

**Table 1.** Workload demands of MB position players for a typical game^*a*^

Avg	Running Distance, m			High-Speed Running Distance, m			High-Speed Running Count			Hard Accelerations			Minutes		Throws		Swings
Game	Total	Defense^*b*^	Baserunning^*b*^	Total^*b*^	Defense^*b*^	Baserunning	Total	Defense^*b*^	Baserunning	Total	Defense^*b*^	Baserunning^*b*^	Defense^*b*^	Baserunning	Total	Hard^*b*^	Total^*b*^
1B	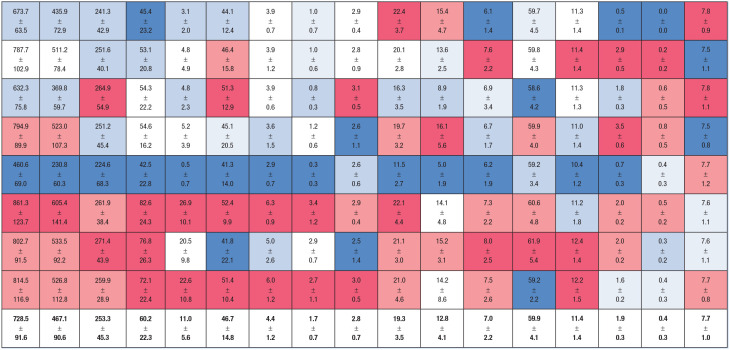																
2B																	
3B																	
SS																	
C																	
CF																	
LF																	
RF																	
**Avg**																	

IQR, interquartile range; MLB, Major League Baseball.

a Data are expressed as the mean ± SD.

b Denotes non-normally distributed data expressed as median ± IQR. Colors indicate very low (dark blue) to very high (dark red).

**Table 2.** Workload demands of MLB position players for a typical 7-day period^*a*^

12	Running Distance (m)			High-Speed Running Distance (m)			High-Speed Running Count			Hard Accelerations			Minutes		Throws		Swings
Avg	Total	Defense^*b*^	Baserunning^*b*^	Total^*b*^	Defense^*b*^	Baserunning	Total	Defense^*b*^	Baserunning	Total	Defense^*b*^	Baserunning^*b*^	Defense^*b*^	Baserunning	Total	Hard^*b*^	Total^*b*^
1B	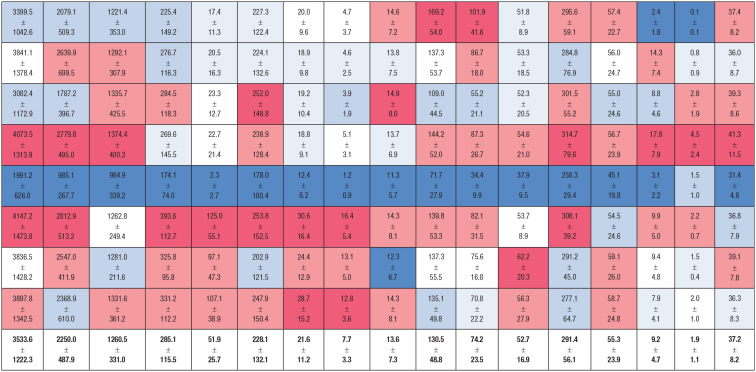																
2B																	
3B																	
SS																	
C																	
CF																	
LF																	
RF																	
**Avg**																	

IQR, interquartile range; MLB, Major League Baseball.

a Data are expressed as the mean ± SD.

b Denotes non-normally distributed data expressed as median ± IQR. Colors indicate very low (dark blue) to very high (dark red).

Total (F_1,7_ = 27.1, *P* < 0.01), high-speed running distance (*P* < 0.01) and high-speed running counts (F_1,7_ = 31.0, *P* < 0.01) were significantly different between positions. Outfielders experienced the greatest running demands, followed by infielders, then catchers. Significant positional differences were also observed for hard accelerations (*F*_1,7_ = 12.9, *P* < 0.01). Interestingly, these were highest among first basemen, followed by outfielders, the remaining infielders, then catchers.

Total throws differed between positions (*F*_1,7_ = 17.7, *P* < 0.01) with middle infielders experiencing the highest demand, followed by outfielders and third basemen, then catchers and first basemen. Hard throws differed between positions (*P* < 0.01) and were highest among second-basemen and shortstops, followed by third-basemen, then outfielders and catchers, followed by first-basemen.

### Discussion

This is the first study to describe the in-game workload demands for position players in MLB across running, throwing, and hitting activities. The data show significant between-position differences for running and throwing metrics. This information may inform the development and delivery of position-specific training programs that (1) prepare healthy athletes for the regular season, (2) allow bench players to maintain chronic loads in-season for an eventual return to the line-up, and (3) better transition players back from injury during the rehabilitation process.

While the workload demands for an average game of MLB are modest compared with other team sports, this study shows that the cumulative workloads arising from multiple games per week within a highly dense schedule create significant load that requires adequate preparation.

Evidence suggests that athletes who are better prepared for the demands of their sport are less at risk of subsequent injury.^13^ High chronic loads (typically defined as the average weekly workload across a 4-week period) as well as reduced spikes in the acute:chronic workload ratio (typically defined as the ratio between the weekly load and the 4-week chronic load) have both been associated with reduced injury risk in other team sports.^6,13,14,18^ The findings of this study set the foundation for development and delivery of training programs that optimize chronic loads and reduce spikes in acute:chronic ratios. By ensuring recovering athletes are conditioned to achieve loads that equal the total load they will typically experience over a 7-day period, the risk of a subsequent spike in workload is reduced upon their transition back into competition. The risk of a workload spike is further reduced if chronic loads above this level are achieved and maintained (eg, a chronic load equivalent to the mean plus 2 standard deviations, as a worst-case scenario) to ensure the athlete is prepared for the majority of possible workload exposures. Importantly, these chronic loads should also include any practice workload the athlete is expected to perform in addition to in-game workload demands.^13^

These findings suggest that players at each position should prepare differently for the demands of the season. For example, outfielders should obtain higher levels of total and high-speed running compared with infielders and catchers; first basemen and outfielders should prepare for a higher degree of hard accelerations; and middle infielders should ensure higher throwing and hard throwing volumes before in-game activity. The goal of this preparation is to expose the athletes to appropriate chronic workloads and thereby build resiliency.

Specific attention should be paid to a host of factors when developing a preseason or return-to-play program, including the position played by the athlete, the density of the playing schedule, and the anticipated playing frequency for the individual athlete. In situations where a player’s position and frequency of play has not yet been determined, we recommend preparing the athlete for the position with the highest demands across each identified metric. For example, one might prepare an infield utility player for the hard throws of a second-baseman, the high-speed running demands of a shortstop, and the hard acceleration demands of a first baseman, to ensure that they are adequately prepared for in-game exposure regardless of position, allowing the manager maximum flexibility in how they can be utilized.

In addition to promoting adequate physical preparation, these data might also provide stimulus to identify occurrences of excessive practice. While there are many desirable reasons to engage in high volumes of practice (such as physiological adaptation, technical skill development, tactical development), the relatively low in-game demands of throwing and hitting presented here might help inform the reconsideration of practice volumes at various times in the year. For example, during in-season periods when fatigue is likely to be higher (due to travel demands, schedule density), and the perceived need for physiological, technical, or tactical development might be lower, these data might help inform how much volume can be removed from the practice environment without compromising game readiness.

The different workload demands may help explain the frequency and type of injuries experienced by players at different positions. Intuitively, we show that high-speed running volumes are highest among outfielders and infielders, who have previously been shown to sustain the most hamstring strains of all player groups (34.6% and 36.0%, respectively).^4^ This aligns with previous research in other team sports linking high-speed running volumes and hamstring strain incidence.^17^ Similarly, calf strains are most common among infielders (38.4%) and outfielders (26.5%), who are shown here to be exposed to the most hard accelerations.^4^ We believe that the data presented here should form the basis for future investigations into associations between workload, injury risk, and positional injury profiles among professional baseball players.

Based on these data, one might reasonably expect that return-to-play timelines could differ by position for a given injury. For example, outfielders need to prepare for a higher degree of high-speed running. A worst-case scenario (which we define as the mean plus 2 standard deviations) for a centerfielder is 619 m of high-speed running over a 7-day period, but only 509 high-speed meters for a second baseman. This additional workload may take longer to accumulate in the rehabilitative process and may result in slightly extended return-to-play timelines for players at this position. Feasibly, to accelerate an athlete’s return-to-play timeline, managers could consider using athletes in other positional roles with lower demands as these workloads are accumulated.

We believe the data presented here can improve individualized rehabilitation after lower limb injuries for players of different positions. Specifically, objective return-to-play criteria can be set using workload data specific to the player’s position. For example, after a hamstring strain injury, position-specific targets can be set for each of the key running metrics identified here, such as total and high-speed running distance, high-speed running counts, and hard acceleration counts. These targets could include both game demands presented here in combination with any expected practice demands. Progress toward these targets can be monitored daily using wearable global positioning system tracking technology, preferably following internal validation across systems to ensure outputs are comparable. While the return-to-play decision-making process is complex and multifactorial in nature, the attainment of these objective workload targets in addition to other criteria (strength, range of motion, etc) may better ensure athletes are adequately prepared for the volume and intensity of game demands, potentially mitigating against the risk of subsequent reinjury.

### Limitations

Statcast data provided to baseball clubs have a number of limitations: (1) whereas the frequency and resolution of the system are known, the standard errors associated with these variables are unknown; (2) the system captures workload experienced by players only while the ball is in play. Consequently, these data may significantly underestimate total workload in a number of cases. Specifically, the total throws made by the catcher in this study is significantly lower than that previously reported,^21^ as many of these throws occur when the ball is no longer in play. Throws made by other position players between innings are also unaccounted for. Total running distance may also be underestimated, as position players are not tracked as they move to and from their positions at the start and end of each inning or as they move to new fielding positions as part of defensive shifts.

Finally, this study sought to define only the in-game workloads experienced by players and does not account for training volumes. While the combination of training and game volumes is required for a complete understanding of total workload experienced by these athletes, this information is beyond of the scope of the current study. Significant challenges currently exist to define total workload for position players across MLB including but not limited to (1) the highly variable and modifiable nature of training workloads, (2) the lack of standardized systems to monitor workload from one team to another, and (3) an unwillingness to share this information in the public domain. We believe it is incumbent on each individual team to solve this problem to ensure training volumes prepare athletes appropriately for the in-game workload demands of MLB.

### Conclusion

These data provide insight into how best to prepare athletes of different positions for the demands of a game, both in terms of preseason preparation and return-to-play benchmarks after injury.
